# Laboratory Validation of a Fully Automated Point-of-Care Device for High-Order Multiplexing Real-Time PCR Detection of Respiratory Pathogens

**DOI:** 10.3390/diagnostics15192445

**Published:** 2025-09-25

**Authors:** Libby C. W. Li, Deborah M. S. Tai, Anita Yee, Nancy B. Y. Tsui, Parker Y. L. Tsang, Sunny L. H. Chu, Chui Ting Leung, Bernice K. W. Leung, Winston Wong, Firaol Tamiru Kebede, Pete Y. M. Leung, Teresa Chung, Cyril C. Y. Yip, Jonathan H. K. Chen, Rosana W. S. Poon, Kelvin K. W. To, Kwok-Yung Yuen, Manson Fok, Johnson Y. N. Lau, Lok Ting Lau

**Affiliations:** 1 Emerging Viral Diagnostics (HK) Limited, Hong Kong, China; 2 Institute for Innovation, Translation and Policy Research, Hong Kong Baptist University, Hong Kong, China; 3 Wu Jieh Yee Institute of Translational Chinese Medicine Research, Hong Kong Baptist University, Hong Kong, China; 4 Department of Industrial and Systems Engineering, The Hong Kong Polytechnic University, Hong Kong, China; 5 Department of Microbiology, The University of Hong Kong, Hong Kong, China; 6 Centre for Virology, Vaccinology and Therapeutics, The University of Hong Kong, Hong Kong, China; 7 Faculty of Medicine, Macau University of Science and Technology, Macau, China; 8 Department of Biology, Hong Kong Baptist University, Hong Kong, China

**Keywords:** point-of-care system, automated genetic pathogen test, multiplex real-time RT-PCR, respiratory tract infection, pathogen detection

## Abstract

**Background/Objectives**: We have previously reported the engineering of a point-of-care (POC) system that fully automates the procedures for nucleic acid extraction and multiplexed real-time RT-PCR, with a major advantage of high-level multiplexing. In this study, we applied and validated the system in a respiratory tract infection setting. **Methods**: An automatic nested real-time RT-PCR assay was developed (POCm). It was a 40-plex assay that simultaneously detected 39 epidemiologically important respiratory pathogens in 1.5 h in the POC system. The analytical and clinical performance was evaluated. **Results**: The analytical sensitivities of the POCm assay were comparable to those of its single-plex counterparts performed manually on a bench-top. The minimum detectable concentrations ranged from 53 copies/mL to 5.3 × 10^3^ copies/mL for all pathogen targets except hCoV-NL63 (5.3 × 10^4^ copies/mL). The quantitative performance was demonstrated by the linear correlations between Ct values and input concentrations for all pathogen targets, with 24 of them demonstrating coefficients of correlation (r) greater than 0.9. The POCm assay was subsequently evaluated in 283 clinical samples. A high level of agreement (98.2–100%) was achieved for pathogen detection results between POCm and standard diagnostic methods. The POCm result was also fully concordant with the result of another commercial POC multiplex platform. For positive clinical samples, pairwise Ct values measured by POCm closely correlated with those of the bench-top reference method (r = 0.70). The feasibility of mutation genotyping of the viral subtype was further demonstrated. **Conclusions**: This study demonstrated the practicality of POCm for routine testing in clinical laboratories. Further clinical trials are being conducted to evaluate the clinical performance of the system.

## 1. Introduction

Pathogen genetic testing is an essential tool to realize a near-to-real-time respiratory infection control, including patient care and epidemic surveillance. Point-of-care (POC) systems can dramatically increase the accessibility of genetic testing in critical low-infrastructure sites such as doctors’ offices, community clinics, and borders [1,2,3]. Lab-on-chip nucleic acid analysis is one of the most promising POC testing methods. By using microfluidic technologies, the entire testing procedure can be miniaturized into a micro-chip, which permits ease of use by minimally trained operators [4]. However, one of the challenges in POC development is the capability to detect a diverse number of potential causative agents in one assay. Acute respiratory tract infection, for example, can be caused by over 20 different strains of viral pathogens [5]. In addition to causative viruses, extra multiplexing capacity is required to discriminate bacteria and fungi that can cause indistinguishable clinical symptoms [5]. The growth of biomarkers developed by next-generation sequencing further necessitates high-order multiplex testing.

To maximize the value of lab-on-chip POC testing, we previously developed an in-house POC system, which was designed to (i) conduct high-order multiplexing with 120 physically separated mini-compartments for real-time PCR; (ii) ensure ease of use with less than 5 min of manual handling; and (iii) incur relatively low manufacturing cost. The whole POC system consists of an enclosed cartridge, where all reactions are carried out, and a microfluidic analyzer for controlling microfluidics and reaction conditions. The development of the automation system and its mechanics has been reported previously [6]. The detection chemistry is based on a multiplexed nested-RT-PCR, followed by a panel of single-plex TaqMan real-time PCRs in individual nano-compartments. The use of PCR methodology provides high flexibility for customizing and updating assay panels from time to time [7,8,9]. It enables rapid, sensitive, and specific detection and characterization of pathogens directly from clinical specimens. It also offers potential for nucleic acid quantification and mutation genotyping.

Nucleic acid amplification tests (NAATs), including PCR, have become an integral part of molecular diagnostics and are used as “standard-of-care” tests for many infectious diseases [10]. Their use in the diagnosis of respiratory infections [11,12], sexually transmitted infections [13], and infectious diarrhea [14] has been recommended in the corresponding guidelines. The ability of NAATs to detect pathogens not recovered by standard culture methods is significant and may aid the diagnosis of pneumonia [15] and bloodstream infections [16]. Miniaturized PCR-based systems are being developed for rapid diagnostics applications. A few of these systems are designed for multiplex detection of up to three or four respiratory pathogen targets [17,18], while the majority focus on single-target detection [19,20]. Some require sample pre-processing, which limits their use outside laboratory settings [21]. Our POCm system surpasses the previous innovations, demonstrating the ability of conducting a 40-plex assay with full automation.

In this study, we evaluated this high-order multiplexing POC system (POCm) in a laboratory setting. Respiratory infection was selected as a model since it is the most common infection globally and is associated with high morbidity and mortality [5,22]. Here, we describe the laboratory assay development, analytical validation, and clinical application of a 40-plex POCm test, which targets 39 pathogen strains that are most relevant to Hong Kong and nearby Asian regions. The test was fully automated with a sample-to-result turnaround time of 90 min. By achieving genetic testing capacity that is simple, rapid, comprehensive, and inexpensive, this POCm platform could fill the unmet needs of patients, healthcare professionals, and epidemiologists in infectious disease control.

## 2. Materials and Methods

### 2.1. POCm Cartridge and Analyzer

All testing procedures are fully automated in the POCm system, which comprises a single-use reaction cartridge and a microfluidic analyzer (Supplementary Figure S1). The cartridge consists of a panel of reaction chambers for nucleic acid extraction, reverse-transcription polymerase-chain reaction (RT-PCR) and real-time PCRs. Primers and/or probes (Integrated DNA Technologies, Coralville, IA, USA) are pre-spotted and dried into relevant chambers during manufacturing. The cartridge further includes multiple reagent reservoirs pre-packaged with buffers and reaction master mixes. The ready-to-use cartridge is stable for a minimum of 6 months when stored at 4 °C. All fluidics within the cartridge are controlled by microchannels and valves interconnecting the reservoirs and chambers. The whole testing procedures are enclosed inside the cartridge. The sealed cartridge is disposed after each test, hence minimizing the risk of contamination by infectious samples and PCR products. The cartridge is manufactured by the injection-molding method, which allows mass production at relatively low cost.

The microfluidic analyzer serves the functions of (i) manipulating the transfer of samples and reagents within the microfluidic network in the cartridge; (ii) controlling reaction temperatures and thermal cycles; and (iii) measuring fluorescent intensities during real-time PCR. It also monitors all reaction parameters and reports any failure during the run. A schematic representation of the validation of the POCm system is shown in Supplementary Figure S2.

### 2.2. POCm Testing Procedures

Three hundred microliters of nasopharyngeal aspirate, endotracheal aspirate, or viral transport medium (VTM) containing swab samples was added to the sample chamber of the cartridge. For deep throat saliva, 0.5 to 1 mL of sample was mixed with 2 mL of VTM, and 300 μL of the diluted sample was added. The cartridge was then loaded into the microfluidic analyzer, and the entire testing workflow was automated in the analyzer. After the run was completed, the analyzer automatically performed data analysis and reported results.

### 2.3. Assay Panel Design

The multiplexed detection panel consisted of 40 targets for 39 respiratory tract disease pathogens, including 26 viruses, 11 bacteria, and 2 fungi (Supplementary Table S1). The pathogens were selected by Department of Microbiology, The University of Hong Kong, based on their epidemiological significance in Hong Kong SAR and nearby regions. Since the assay was designed during the early outbreak of COVID, two detection targets were designed for SARS-CoV-2 in order to enhance the detection confidence. Enterovirus and rhinovirus were not included in this evaluation study since the POCm assay cannot confidently distinguish between them. Further assay optimization is being carried out, and the two viruses will be included in the next version of the POCm assay.

Three quality control assays were further included in the panel. They were *glyceraldehyde 3-phosphate dehydrogenase (GAPDH)* as the sample quality control, *schizosaccharomyces pombe* RNA (*SUC1*) (ATCC, Manassas, VA, USA; Cat# 24843) which was spiked-in during each run for experimental processing control, and a non-pathogen target for real-time PCR control (GenScript, Nanjing, China).

The assay was based on nested real-time RT-PCR. It comprised a multiplexed one-step RT-PCR for 40 pathogen targets and 2 controls (*GAPDH* and *SUC1*), i.e., a 42-plex reaction. It was followed by an array of single-plex real-time PCRs carried out in 120 individual mini-chambers for the 40 pathogen targets and the 3 controls. Outer primers for RT-PCR were designed with melting temperature (T_m_) ranging from 67 °C to 76 °C in order to achieve optimal amplification specificity. Each real-time PCR assay consisted of a pair of inner primers and a TaqMan probe. The T_m_ of inner primers was 59–66 °C. Primers of each target were tested individually using conventional manual methods to evaluate the amplification conditions. The primers of all the targets were then pooled together to test for any interference. If the Ct value of a target in the multiplex is much higher than that of the single-plex reaction, the corresponding primers are re-designed.

### 2.4. PCR

The first-round RT-PCR master mix was prepared using Path-ID Multiplex One Step RT-PCR Kit (Thermo Fisher Scientific, Waltham, MA, USA; Cat# 4442136). The RT-PCR chamber of the cartridge was pre-spotted with 42 pairs of outer primers, which were re-suspended in master mix to a final concentration of 0.07 μM. The entire volume of extracted nucleic acids (16 μL) was used for the reaction. RT was carried out at 48 °C for 10 min, 95 °C for 10 min, followed by 20 PCR cycles of 95 °C for 15 s and 65 °C for 45 s. The final PCR product was automatically 66.7-fold diluted with nuclease-free water.

**Figure 1:**
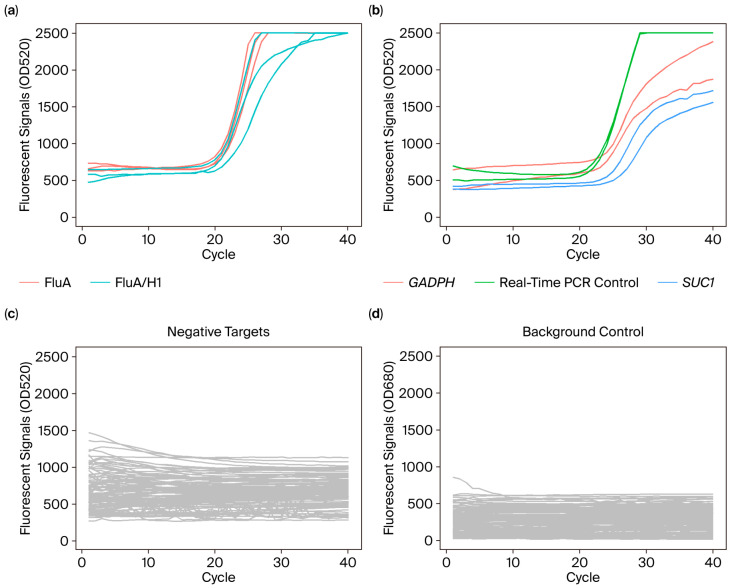
Example amplification plots of a patient sample as tested by POCm. Fluorescence signals of the 40 pathogen targets and 3 quality controls were obtained in a single run. The data were separated into four graphs for interpretation. (**a**) The sample tested positive for Flu A/H1. Positive amplification signals were detected for Flu A generic assay (red line) and H1 subtype specific assay (green line). The qPCR curves showed typical sigmoidal shape. The real-time PCR assays were performed in triplicate. (**b**) Amplification plots for internal control (*GAPDH*) (red line), external control (*SUC1*) (blue line) and real-time PCR control (green line). Duplicated real-time PCR reactions were performed for each control assay. (**c**) Negative result was obtained for all of the 38 non-target assays (grey line), indicating their assay specificities. (**d**) Atto 665 fluorescent signals (grey line) for background monitoring throughout real-time PCR.

The second-round real-time PCR master mix was prepared using Premix Ex Taq (Probe qPCR) Kit (Takara Bio, Shiga, Japan; Cat# RR390). Atto 665 fluorescent dye (Sigma-Aldrich, Burlington, MA, USA; Cat #93711) was added in a concentration of 0.03 μM for background monitoring. Each real-time PCR mini-chamber in the cartridge was pre-spotted with a pair of inner primers and probe for one detection target. Each pathogen target or control was detected in duplicate or triplicate (Supplementary Table S2). The primers and probe were re-suspended in master mix to final concentrations of 0.5 μM and 0.2 μM, respectively. Real-time PCR was carried out at 95 °C for 30 s, followed by 40 cycles of 96 °C for 1 min and 55 °C for 45 s. Figure 1 shows an example of amplification results from a sample positive for Flu A/H1.

### 2.5. Manually Performed RT-PCR Tests

The detection assays were optimized by conventional manual methods using bench-top instruments before adopting POCm. Bench-top manual nucleic acid extraction was performed using QIAamp MinElute Kit (Qiagen, Valencia, CA, USA; Cat# 57704). First-round RT-PCR and second-round real-time PCR were carried out in Veriti 96-Well Thermal Cycler and QuantStudio 7 Flex Real-Time PCR System (both from Thermo Fisher Scientific, Waltham, MA, USA), respectively, with conditions as described above.

### 2.6. Preparation of Reference Controls

To evaluate the test performance, pathogen-derived genomic DNA and RNA (ATCC, Manassas, VA, USA), PCR controls (Vircell, Granada, Spain) or plasmids with inserts of target sequences (GenScript, Nanjing, China) were used for constructing reference control plasmids (Supplementary Table S3). The plasmids were used for evaluation tests of all bacterial and fungal targets, as well as DNA viruses including AdV and BoV. For RNA viruses, in vitro transcriptions were further performed to generate reference control RNAs that contained the RT-PCR amplicon sequences.

Twelve mixtures of reference controls were further prepared (Supplementary Table S4). For 11 of the mixtures, three to four pathogen controls were spiked in a background of human RNA extracted from HeLa cells. The 12th mixture served as a negative control with human RNA only.

### 2.7. Evaluation with Clinical Samples

The POCm assay was further evaluated using archived clinical specimens retrieved by the Department of Microbiology, Queen Mary Hospital, from January 2020 to December 2021. A total of 283 samples were tested. The samples included nasopharyngeal aspirate (NPA) (*n* = 141), nasopharyngeal swab (NPS) (*n* = 136), combined nasopharyngeal and throat swab (*n* = 1), endotracheal aspirate (*n* = 1), and saliva (*n* = 4) from patients suffering from influenza-like signs and symptoms (Supplementary Table S5). They were previously tested with “standard-of-care” tests, which included immunofluorescent antibody assays (IFA) (D3 Ultra 8 DFA Respiratory Virus Screening and ID Kit, QuidelOrtho, San Diego, CA, USA; Cat# I-01-110000), conventional PCR, Xpert Xpress Flu/RSV (Cepheid, Sunnyvale, CA, USA; Cat# GXFLU/RSV-CE-10) and/or FilmArray Respiratory Panel (bioMérieux, Marcy-l’Étoile, France; Cat# RFIT-ASY-0124). For pathogens not covered by IFA and Xpert Xpress Flu/RSV, further tests were conducted by the reference laboratory (Public Health Laboratory Centre in Hong Kong) using in-house multiplex assays targeting respiratory viruses. Details on the standard-of-care tests for each pathogen are listed in Supplementary Table S6.

### 2.8. Statistical Analysis

Statistical analyses were conducted using SigmaPlot 13 (Systat Software, Inc., San Jose, CA, USA) and R (version 4.3.1) (The R Foundation, Ames, IA, USA).

## 3. Results and Discussion

### 3.1. Selection of Respiratory Pathogens for Multiplex Assay Panel

The selection of respiratory pathogens for inclusion in our assay panel (Supplementary Table S1) was guided by both clinical relevance and epidemiological significance. Definite pathogens, including all the targeted respiratory viruses, *Mycobacterium tuberculosis* (*MTB*), and *Burkholderia pseudomallei,* etc., were included due to their established roles as causative agents. Notably, *MTB* was incorporated based on increasing evidence supporting the use of upper respiratory tract specimens for diagnosis of tuberculosis, with a sensitivity reaching 81.8% [23]. In addition, *Pneumocystis jirovecii* (*PJ*) was included in the panel, despite potentially indicating colonization, as its detection in NPA specimens may guide further testing with bronchoalveolar lavage in patients who are suspected to have *Pneumocystis* pneumonia [24]. Furthermore, our panel also encompassed bacteria such as *Streptococcus pneumoniae*, *Staphylococcus aureus*, and *Streptococcus pyogenes*. Although their detection in the upper respiratory tract specimens may represent asymptomatic colonization, this information can be relevant to decisions on infection control or prevention measures. Clinicians can use clinical information for treatment decisions, including the use of antibiotics. Our broadened panel covering these additional pathogens would be able to enhance diagnostic yield and inform more comprehensive patient management.

### 3.2. Analytical Detectability

The microfluidics layout of the POCm cartridge was designed in order to elevate the multiplexing capability. Firstly, the starting amount of nucleic acids is an important factor for detection sensitivity. Inside the POCm cartridge, the whole extracted volume of nucleic acids (16 μL) was channeled for the first-round RT-PCR. On the other hand, only 1/20 volume of extracted nucleic acids (5 μL out of 100 μL) could be used for the bench-top approach. Secondly, for the second-round real-time PCR detection, single-plex reactions were individually carried out in 120 physically separated chambers in the POCm cartridge. This design could reduce cross-reactivity among different pathogen assays and therefore enhance detection specificity. Owing to the high-multiplexing capacity, more than one PCR target can be designed for a particular pathogen. This increases detection reliability for epidemiologically or genetically complex pathogens. To evaluate the benefit of the POCm platform over the bench-top approach for high-order multiplexing reactions, their analytical performance was compared.

The analytical detectability of the targeted pathogens was compared between POCm and the manual approach by using the limiting dilution method. Reference controls of individual pathogens (Supplementary Table S3) were serially diluted and were tested in triplicate at each concentration. The lowest concentration at which all of the triplicated reactions showed positive results was referred to as the minimum detectable concentration (MDC), which was expressed as copies per milliliter of sample (copies/mL).

**Figure 2:**
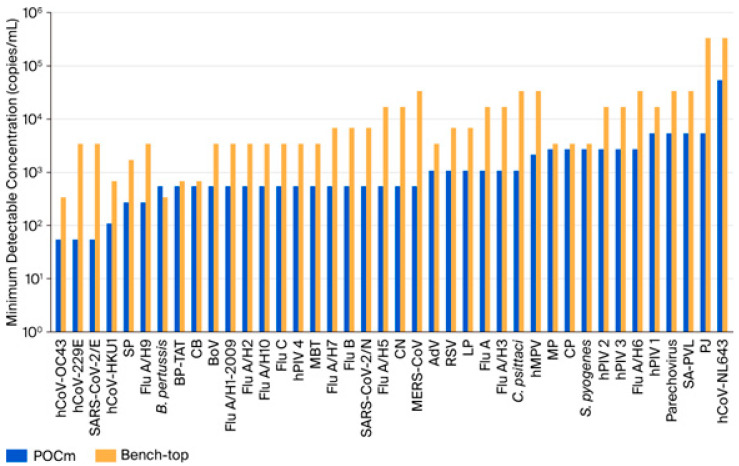
Comparison of analytical sensitivities between POCm and bench-top methods. POCm (blue bar) showed similar or better analytical sensitivities (lower minimum detectable concentrations) than the bench-top method (orange bar) for all pathogen targets except *B pertussis*.

Multiplex assays commonly showed decreased detection sensitivities when compared to their single-plex counterparts. This phenomenon was also observed in our respiratory pathogen assays that were carried out using the manual bench-top platform. When switching from manual-single-plex to manual-40-plex reactions, the detectability of the 40 pathogen targets dropped. Their MDCs were significantly increased for a median of 11-fold (*p* < 0.05, Wilcoxon signed-rank test) (Table 1). On the other hand, we showed that this shortcoming of multiplexing was resolved by the POCm platform. For all 40 pathogen targets, the detectability of the automatic-POCm assay was restored to that of the manual-single-plex counterparts with no statistically significant difference in MDCs (*p* = 0.562, Wilcoxon signed-rank test). The MDCs of 53 to 5300 copies per milliliter of sample (copies/mL) were obtained for all pathogen targets except hCoV-NL63 (53,000 copies/mL) (Table 1 and Figure 2). 

To inspect cross-reactivity of the POCm 40-plex reaction, we analyzed the reference control mixtures (Supplementary Table S4) at concentrations 1000-fold higher than the corresponding MDCs. Negative results were obtained for all non-targeted pathogens. Hence, no cross-reactivity among the pathogen targets was observed for POCm.

### 3.3. Linearity

**Figure 3:**
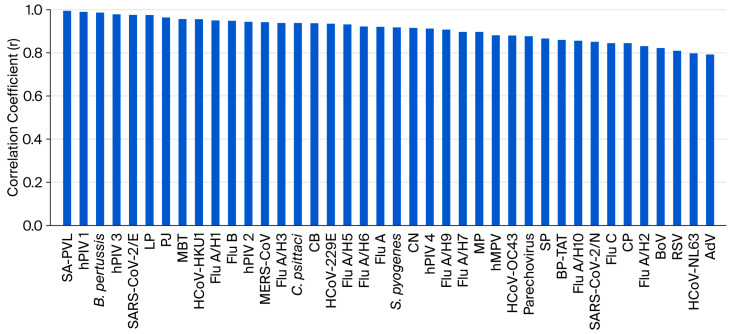
Evaluation of quantitative performance of POCm. For all of the 40 pathogen targets, linear relationships were obtained between Ct values and log (concentrations in copies/reaction) as measured by POCm (*p* < 0.05). Coefficients of correlation (r) ranged from 0.792 to 0.994. Twenty-four of the pathogen targets showed r > 0.9.

The capability of the POCm platform in quantification of respiratory pathogens was evaluated. Linearity panels of the 40 pathogen targets were generated using the reference control mixtures (Supplementary Table S4). The mixtures were serially log_10_ diluted from 1000- to 1-fold of MDCs of the corresponding pathogen targets and analyzed by POCm. We found statistically significant correlations between Ct values and input concentrations for all of the 40 pathogen targets (*p* < 0.05, ANOVA for linear regression). The coefficients of correlation (r) ranged from 0.792 to 0.994, with 60% (24 of 40) of the targets showing r > 0.9 (Figure 3). The result hence demonstrated that POCm can potentially be used for pathogen load estimation.

### 3.4. Clinical Sample Evaluation

The clinical evaluation of POCm was carried out in the laboratory of the Department of Microbiology, Queen Mary Hospital. A total of 283 clinical samples were tested and included for clinical performance analysis. This study involved POCm assays of two initial versions (11-plex and 18-plex assays) (Supplementary Table S5) and a final 40-plex assay (Supplementary Table S1). The results from all of the POCm assay versions were combined for analysis.

Of the 39 pathogens that the POCm assay targeted, 13 pathogens tested positive in the “standard-of-care” tests on at least 1 out of the 283 samples (Supplementary Table S7). Eleven of the pathogens that were tested on ≥200 clinical samples were further evaluated for testing performance (Table 2). In general, for 10 of the 11 (90.91%) pathogens, the results of POCm highly agreed with those diagnosed by standard-of-care tests, with agreements and Cohen’s κ coefficients achieving 98.2–100% and 94.6–100%, respectively. The detection of *Mycoplasma pneumoniae* (MP) was sub-optimal since POCm could only detect one of the two samples that tested positive by the standard-of-care test (Table 2). Further evaluation of the MP detection performance with additional positive samples is required.

We further evaluated the clinical accuracy of the POCm platform. The sample sizes of all of the 11 pathogens were sufficient for specificity calculation [25]. The specificities were 100% for all of the assays (Table 2). For sensitivity evaluation, a minimum sample size of 200 with at least 20 positive samples has been recommended for diseases with 5–10% prevalence [25,26]. We therefore selected Flu A (Matrix), Flu A (H1-pdm09) and SARS-CoV2, which had sufficient sample sizes, for sensitivity calculation. The detection sensitivities were 89.8%, 95.2% and 91.7% for Flu A (Matrix), Flu A (H1-pdm09) and SARS-CoV2, respectively (Table 2). Despite no definite threshold requirement, the detection sensitivities should be further improved in order to have better utilization in frontline diagnostics. Potential assay optimization strategies include improving binding buffer and elution buffer formulation to recover more templates during nucleic acid extraction, increasing the amount of input in the RT-PCR step, constantly reviewing any mutations in the gene targets that may affect the detection and redesign primers/probe if necessary.

In addition to the use of standard-of-care testing results as a reference, we also compared the performance of POCm with another POC system manufactured by FilmArray [27], which is one of the multiplexing POC platforms available on the market. FilmArray provides qualitative detection of 23 pathogen strains with its Respiratory Panel (BioFire FilmArray Panel, bioMérieux, Marcy-l’Étoile, France). We tested ten positive clinical samples, as well as three negative samples collected from healthy individuals in parallel using both POCm and FilmArray. As shown in Table 3, the results obtained from both POC systems were fully concordant.

In summary, the validation results of clinical samples demonstrated that the performance of POCm concurred with those of standard-of-care testing methods as well as other POC platforms on the market. Due to the limiting sample size, we could not fully evaluate the clinical performance of all of the 39 pathogens. In addition, for some of the pathogens, the studied clinical samples were not the representative sample types when clinically indicated, which might influence result accuracy. Nonetheless, the promising results of this study will promote the future expansion of clinical studies in terms of sample sizes and testing sites.

### 3.5. Pathogen Co-Infection Detection

One advantage of the multiplexed detection platform is pathogen co-infection identification [28,29]. During the period of this study, one clinical sample, 20QM0123, was initially diagnosed as single-pathogen infection with SARS-CoV-2. With the use of POCm, we identified additional co-infected pathogen hMPV in these samples (Table 4), which was subsequently confirmed by standard-of-care tests. We also detected co-infection in one sample (20QM0113), which was concordant with the initial co-infection diagnostic result (Table 4).

Two additional samples, 20QM0119 and 21QM0003, were diagnosed with SARS-CoV-2 and RSV, respectively, but *Streptococcus pneumoniae* (*SP*) was also detected. *SP* may usually be considered as normal colonization in the upper respiratory tract. Hence, we demonstrated that POCm could provide a more comprehensive pathogen screening, as well as the potential of our assays to detect colonization such as *SP* in NPS/NPA samples, which may assist in more informative targeted patient care [30].

### 3.6. Measurement Agreement of Ct Values

**Figure 4:**
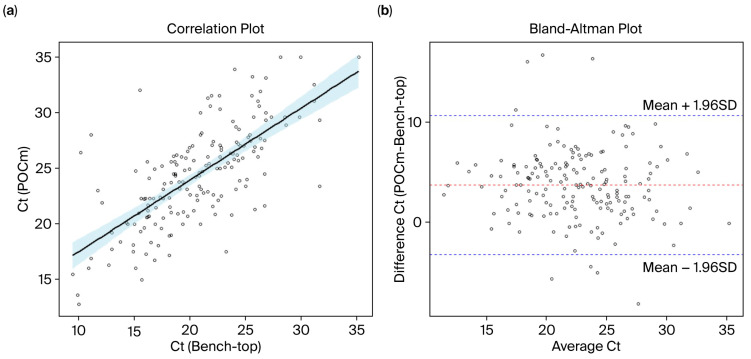
Ct value agreement between POCm and bench-top methods. (**a**) Scatter plot of Ct values as measured by POCm and bench-top methods. The Ct values obtained by both methods were significantly correlated (*p* < 0.05, r = 0.70, Pearson’s correlation test). (**b**) Bland–Altman plot was used to evaluate the Ct value agreement between POCm and the bench-top method. There were systematically higher Ct values for POCm when compared with the corresponding bench-top result (bias = 3.64) (red dashed line). The blue dashed lines represent 95% limits of agreement.

We further assessed the performance of POCm among the clinical samples. A total of 114 clinical samples that tested positive using POCm were evaluated using the bench-top approach in parallel, and the measurement agreement of Ct values was analyzed. We found a statistically significant correlation of overall Ct values between POCm and the bench-top tests (*p* < 0.05, r = 0.70, Pearson’s correlation test) (Figure 4a). Bland–Altman analysis showed systematically higher Ct values for POCm when compared with the corresponding bench-top result (bias = 3.64) (Figure 4b). Despite the bias, 95% of the data points fell within the limits of agreement (−3.37–10.65) (Figure 4b). The result hence confirmed that nucleic acid measurement by POCm was comparable to that of the reference bench-top approach. In practice, whether the bias would have any clinical implications, such as affecting the detection sensitivity in particular for samples with low pathogen loads, still warrants further investigation.

### 3.7. Mutation Genotyping

**Figure 5:**
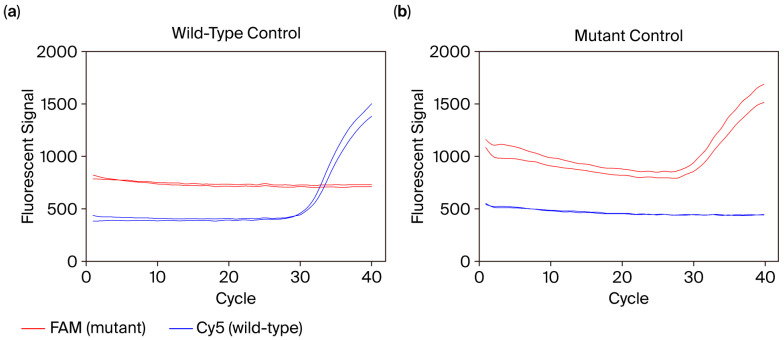
Example amplification plots of allelic discrimination assay for the mutation del:21765:6 of SARS-CoV-2. The allelic discriminative TaqMan probes for the mutant and wild-type alleles were labeled with FAM and Cy5 fluorescent dyes, respectively. The performance of allelic discrimination was confirmed by using synthetic oligonucleotides containing the (**a**) wild-type and (**b**) mutant alleles.

Mutations acquired by emerging pathogens are valuable biomarkers for differentiating virulent pathogen strains and tracing epidemic transmission events. We therefore assessed the feasibility of mutation genotyping by POCm. As a feasibility evaluation, a TaqMan allelic discrimination assay was designed to genotype del:21765:6 mutation in the gene coding spike protein of SARS-CoV-2, a deletion found in the alpha variant [31,32]. The assay involved a pair of TaqMan probes that were 5′-labeled with Cy5 and FAM to discriminate wild-type and mutant alleles, respectively (Integrated DNA Technologies, Coralville, IA, USA). Synthetic oligonucleotides with amplicon sequences of the wild-type and mutated pathogen strains (Integrated DNA Technologies, Coralville, IA, USA) were used for testing. Figure 5 shows the amplification result of POCm, with genotypes of the wild-type and mutant control oligonucleotides being correctly detected. Since the POCm cartridge can simultaneously perform up to 120 real-time PCRs in individual mini-chambers, a large panel of mutations can be genotyped in a single automated test. This is favorable for pathogens with abundant variants such as SARS-CoV-2. Moreover, assay design and evaluation are straightforward, which allows rapid adaptation to newly emerging variants. This would facilitate the development of comprehensive assays for both detection and sub-typing of fast-evolving pathogens.

## 4. Conclusions

The POCm system that we previously developed offers a major advantage to infectious disease diagnostics, as it can detect a large panel of pathogen infections in a single automated test. In this study, we demonstrated its practicality in respiratory tract infections, including influenza and other concurrent pathogens that share similar clinical symptoms but require distinct disease treatment and management [5].

In this study, we developed a 40-plex real-time RT-PCR assay that targeted 39 pathogens. The order of multiplexing is, to our knowledge, the largest among the POC platforms available on the market [3,33]. The assay was demonstrated to be efficiently automated in the POCm system, with analytical performance comparable to that of the bench-top method. The fully integrated POCm system with automated nucleic acid extraction and multiplex real-time RT-PCR was evaluated with clinical samples. The detection results were highly concordant with those of standard diagnostic methods, as well as a commercially available RT-PCR-based POC platform. With the advantage of high-order multiplexing, POCm was able to detect a co-infection that had been missed by standard diagnostic methods. This result hence highlights the benefit of POCm in the comprehensive identification and exclusion of pathogens, as well as in the detection of mixed-pathogen infections [28,34,35]. In addition, the demonstrated semi-quantitative capability of POCm suggests a potential application for the detection and complementary quantification of pathogen loads, which has been suggested to be associated with disease severity and patient outcomes [36,37].

The current POCm test allowed for subtyping of common pathogens, such as influenza A, by including both a generic assay and various assays specific to the subtypes. POCm could potentially add another level of pathogen characterization by genotyping mutations acquired by rapidly emerging strains [31,32]. As a feasibility demonstration, we were able to genotype an alpha-variant mutation in SARS-CoV-2 by using a POCm allelic-discriminative assay. Along with rapidly sequenced pathogen genomes, POCm would allow prompt development of molecular assays that match the pathogen strains responsible for the outbreak [7,8,9].

In conclusion, POCm is both robust and reliable for high-order multiplexed pathogen detection. It could aid infection diagnosis when utilized in conjunction with clinical history and epidemiological information. With straightforward real-time RT-PCR primer design, the detection targets of POC assays can be promptly updated to accommodate both existing and newly emerging pathogens in one test [38]. Combined with the relative simplicity and low production cost of the single-use cartridge, the POCm system has the potential to streamline clinical diagnostics as well as public health surveillance in the fight against recurrent and upcoming unforeseen epidemics. Further large-scale clinical studies will be valuable in order to evaluate various performance parameters, including clinical sensitivities, specificities and predictive values. Performance studies under different environmental conditions are worthwhile to assess the robustness of the POCm system outside well-equipped laboratories. In addition, guidelines for system operation and training should be established, and usability tests should be conducted to evaluate suitability in community and field use.

## Figures and Tables

**Table 1:** Comparison of minimum detectable concentrations and corresponding Ct values between the bench-top and POCm methods.

Pathogens	Bench-Top					POCm 40-Plex		Fold Change
	Single-Plex ^a^		40-Plex ^a^		Fold Change			
	Minimum Detectable Concentration ^b^	Ct	Minimum Detectable Concentration ^a^	Ct	40-plex/ Single-Plex	Minimum Detectable Concentration ^a^	Ct	(POCm 40-Plex/Bench-Top 40-Plex)
	(Copies/mL)	(Median) ^c^	(Copies/mL)	(Median) ^b^		(Copies/mL)	(Median) ^b^	
AdV	333	33.28	3333	35.66	10.00	1067	29.73	3.20
BoV	3333	27.08	3333	28.17	1.00	533	25.99	0.16
hCoV-229E	333	24.97	3333	24.04	10.00	53	25.32	0.16
hCoV-HKU1	667	24.05	667	25.52	1.00	107	25.8	0.16
hCoV-NL63	33,333	28.11	333,333	28.06	10.00	53,333	25.02	1.60
hCoV-OC43	333	27.53	333	24.83	1.00	53	26.98	0.16
Flu A	333	26.66	16,667	25.21	50.00	1067	26.55	3.20
Flu A/H1-2009	333	23.97	3333	24.04	10.00	533	27.15	1.60
Flu A/H2	333	28.94	3333	29.1	10.00	533	27.45	1.60
Flu A/H3	333	26.1	16,667	26.96	50.00	1067	27.05	3.20
Flu A/H5	3333	26.59	16,667	25.36	5.00	533	26.89	0.16
Flu A/H6	3333	25.79	33,333	25.45	10.00	2667	26.78	0.80
Flu A/H7	667	26.19	6667	28.2	10.00	533	27.77	0.80
Flu A/H9	333	22.98	3333	27.16	10.00	267	28.03	0.80
Flu A/H10	333	29.41	3333	30.29	10.00	533	29.07	1.60
Flu B	333	27.49	6667	27.52	20.00	533	26.43	1.60
Flu C	333	27.16	3333	27.43	10.00	533	25.97	1.60
MERS-CoV	3333	26.99	33,333	25.24	10.00	533	26.4	0.16
hMPV	667	26.26	33,333	23.86	50.00	2133	27.73	3.20
hPIV 1	3333	29.58	16,667	26.07	5.00	5333	25.37	1.60
hPIV 2	3333	24.02	16,667	25.25	5.00	2667	26.66	0.80
hPIV 3	3333	25.86	16,667	26.23	5.00	2667	27.23	0.80
hPIV 4	667	26.02	3333	27.07	5.00	533	26.9	0.80
Parechovirus	3333	27.17	33,333	28.52	10.00	5333	27.87	1.60
RSV	333	27.37	6667	29.32	20.00	1067	25.4	3.20
SARS-CoV-2/E	333	20.98	3333	25.56	10.00	53	24.55	0.16
SARS-CoV-2/N	667	28.2	6667	27.28	10.00	533	24.7	0.80
*MTB*	333	25.97	3333	24.15	10.00	533	22.83	1.60
*MP*	3333	25.77	3333	24.32	1.00	2667	24.79	0.80
*LP*	6667	24.31	6667	23.1	1.00	1067	25.73	0.16
*B pertussis*	333	27.53	333	27.83	1.00	533	25.07	1.60
*C psittaci*	6667	30.35	33,333	26.24	5.00	1067	25.72	0.16
*BP-TAT*	667	26.94	667	26.45	1.00	533	24.13	0.80
*CB*	667	28.41	667	27.61	1.00	533	25.67	0.80
*CP*	3333	28.36	3333	26.21	1.00	2667	24.6	0.80
*SA-PVL*	3333	26.72	33,333	26.99	10.00	5333	25.92	1.60
*SP*	333	28.99	1667	25.56	5.00	267	23.73	0.80
*S pyogenes*	3333	27.18	3333	26.48	1.00	2667	23.53	0.80
*CN*	333	22.88	16,667	25.22	50.00	533	26.05	1.60
*PJ*	33,333	19.33	333,333	19.03	10.00	5333	22.17	0.16

^a^ In single-plex, only the primer set of the pathogen target under evaluation is used in real-time RT-PCR reactions. In 40-plex, primers of all the 40 pathogen targets are combined in one single real-time RT-PCR reaction while each target is evaluated separately. ^b^ Minimum detectable concentrations refer to the lowest concentrations of reference controls that were consistently detected in all of the triplicated real-time RT-PCR runs. ^c^ Median Ct values of the triplicated real-time RT-PCR runs.

**Table 2:** Clinical performance of POCm in detecting respiratory pathogens.

Target	Diagnosis ^a^		POCm Result		Sensitivity	Specificity	Agreement ^b^	Concordance ^c^	PPV ^d^	NPV ^d^
	Result	n	Positive (*n* ^e^)	Negative (*n* ^e^)	% (95% CI) ^f^	% (95% CI)	% (95% CI)	% (95% CI)	% (95% CI)	% (95% CI)
AdV	Positive	3	3	0	Not analyzed ^g^	100	100	100	100	100
	Negative	274	0	274		(98.6–100)	(98.6–100)	(100–100)	-	(98.6–100)
Flu A	Positive	49	44	5	89.8	100	98.2	93.6	100	97.9
	Negative	234	0	234	(78.2–95.6)	(98.4–100)	(95.9–99.2)	(88.0–99.1)	(92.0–100)	(95.2–99.1)
Flu A/H1	Positive	42	40	2	95.2	100	99.3	97.1	100	99.2
	Negative	239	0	239	(84.2–98.7)	(98.4–100)	(97.4–99.8)	(93.2–100.0)	(91.2–100)	(97.0–99.8)
Flu A/H3	Positive	7	7	0	Not analyzed	100	100	100	100	100
	Negative	276	0	276		(98.6–100)	(98.7–100)	(100–100)	(64.6–100)	(98.6–100)
Flu B	Positive	10	9	1	Not analyzed	100	99.6	94.6	100	99.6
	Negative	273	0	273		(98.6–100)	(98.0–99.9)	(83.9–100)	(70.1–100)	(97.9–99.9)
hMPV	Positive	5	5	0	Not analyzed	100	100	100	100	100
	Negative	272	0	272		(98.6–100)	(98.6–100)	(100–100)	(56.6–100)	(98.6–100)
hPIV1	Positive	8	8	0	Not analyzed	100	100	100	100	100
	Negative	269	0	269		(98.6–100)	(98.6–100)	(100–100)	(67.6–100)	(98.6–100)
hPIV3	Positive	11	11	0	Not analyzed	100	100	100	100.0	100
	Negative	266	0	266		(98.6–100)	(98.6–100)	(100–100)	(74.1–100)	(98.6–100)
RSV	Positive	10	9	1	Not analyzed	100	99.6	94.6	100	99.6
	Negative	267	0	267		(98.6–100)	(98.0–99.9)	(83.9–100)	(70.1–100)	(97.9–99.9)
SARS-CoV-2	Positive	24	22	2	91.7	100	99.3	95.3	100	99.2
	Negative	253	0	253	(74.2–97.7)	(98.5–100)	(97.4–99.8)	(88.7–100)	(85.1–100)	(97.2–99.8)
*Mycoplasma* *pneumoniae (MP)*	Positive	2	1	1	Not analyzed	100	99.6	66.5	100	99.6
	Negative	275	0	275		(98.6–100)	(98.0–99.9)	(0.97–100)	-	(98.0–99.9)

^a^ Samples were previously diagnosed by “standard-of-care” test including immunofluorescent antibody assay, conventional PCR, GeneXpert and/or Filmarray (see *Materials and Methods* for details). ^b^ Agreement = (TP + TN)/(TP + FP + TN + FN), where TP, true-positive; TN, true-negative; FP, false-positive; FN, false-negative. ^c^ The Cohen’s κ coefficient calculation was used to estimate the concordance and the corresponding confidence interval. ^d^ PPV, positive predictive value; NPV, negative predictive value. ^e^ Variations in total number of samples for each pathogen were due to their absence in the initial version of POCm assays. ^f^ 95% confidence interval was calculated using Wilson score method. ^g^ Sensitivity was not analyzed due to insufficient number of positive samples.

**Table 3:** Concordant clinical result of 13 samples as determined by FilmArray and the POCm system.

Sample	Specimen Type ^a^	Diagnosed Pathogen	POCm		FilmArray	Agreement
			Target	Ct Value	Target	
20QM0214	NPA	Flu A/H1	Flu A (matrix)	18.03	Flu A/H1	Concordant
			Flu A/H1	17.28		
20QM0055	NPS	Flu A/H1	Flu A (matrix)	21.16	Flu A/H1	Concordant
			Flu A/H1	20.99		
20QM0176	NPA	Flu A/H3	Flu A (matrix)	23.76	Flu A/H3	Concordant
			Flu A/H3	23.75		
21QM0002	NPA	Flu B	Flu B	25.90	Flu B	Concordant
20QM0219	NPS	hPIV1	hPIV1	26.87	hPIV1	Concordant
20QM0175	NPA	hPIV3	hPIV3	22.23	hPIV3	Concordant
20QM0062	NPA	hMPV	hMPV	18.94	hMPV	Concordant
20QM0153	NPS	RSV	RSV	23.72	RSV	Concordant
20QM0042	NPA	AdV	AdV	24.76	AdV	Concordant
20QM0212	NPA	*MP*	*MP*	22.11	*MP*	Concordant
20M048	NPS	Normal control	Negative	-	Negative	Concordant
20M049	NPS	Normal control	Negative	-	Negative	Concordant
20M057	NPS	Normal control	Negative	-	Negative	Concordant

^a^ NPA, nasopharyngeal aspirate; NPS, nasopharyngeal swab.

**Table 4:** POCm results of co-infected samples.

Sample	Specimen Type ^a^	Diagnosed Pathogens	POCm	
			Positive Target	Ct Value
20QM0123 ^b^	NPS	SARS-CoV-2, hMPV	SARS-CoV-2/E	25.48
			SARS-CoV-2/N	27.69
			hMPV	29.30
20QM0113	NPA	Flu A/H1, hPIV4	Flu A	19.04
			Flu A/H1	18.34
			hPIV4	23.12

^a^ NPA, nasopharyngeal aspirate; NPS, nasopharyngeal swab. ^b^ Samples were initially diagnosed as single-pathogen infections. Co-infections were subsequently confirmed by standard diagnostic methods.

## Data Availability

The data presented in this study are available on request from the corresponding author.

## Supplementary Materials

The following supporting information can be downloaded at: https://www.mdpi.com/article/10.3390/diagnostics15192445/s1, Figure S1: POCm cartridge and microfluidic analyzer; Figure S2: Schematic diagram of the current study; Table S1: Pathogen targets of the respiratory infectious disease assay panel; Table S2: Content of real-time PCR mini-chambers in POCm cartridge for respiratory disease pathogen detection; Table S3: Construction of reference controls for evaluation tests; Table S4: Reference control mixtures constructed from reference controls of different pathogens; Table S5: Information of pre-clinical study of POCm in Department of Microbiology, Queen Mary Hospital, Hong Kong; Table S6: Standard-of-care tests of the 39 selected pathogens in Department of Microbiology, Queen Mary Hospital, Hong Kong; Table S7: Clinical evaluation of the 39 pathogen targets separated by the three phases.
