# Supplementary material for: Laboratory Validation of a Fully Automated Point-of-Care Device for High-Order Multiplexing Real-Time PCR Detection of Respiratory Pathogens
Source: Diagnostics (Basel). 2025 Sep 25;15(19):2445. doi: 10.3390/diagnostics15192445 (PMC12523385; doi:10.3390/diagnostics15192445)
Supplement: Supplementary file 1 [file diagnostics-15-02445-s001.zip › diagnostics-3821562-supplementary.pdf]

Laboratory Validation of a Fully Automated Point-of-Care Device for High-Order Multiplexing Real-Time PCR Detection of Respiratory Pathogens

Corresponding author: Lok Ting Lau (terencelau@hkbu.edu.hk)

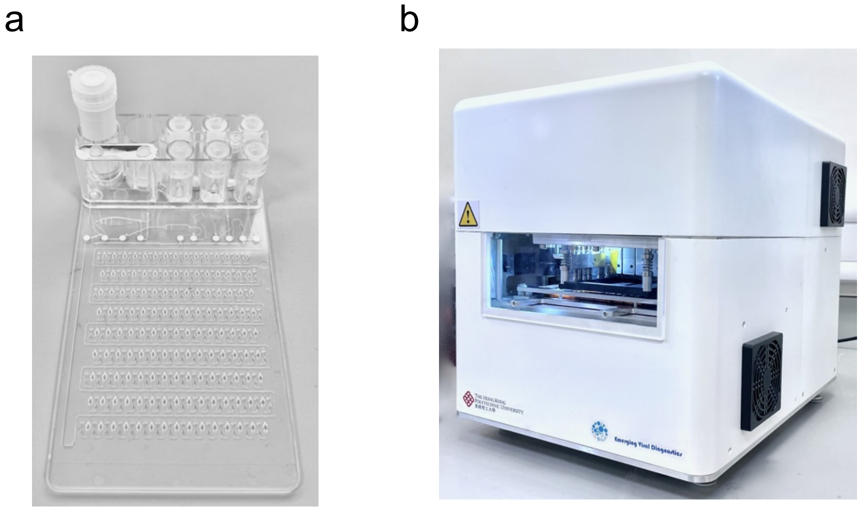

Supplementary Figure S1. POCm cartridge and microfluidic analyzer.

(a) The cartridge (Dimension of Chip: 8.5 x 15.2 cm WD; Reagent tank: 8.4 x 2.8 x 3cm WDH) comprises multiple pre-packaged reagent reservoirs, reaction chambers and real-time PCR mini-chambers pre-spotted with primers and probes. The reservoirs and chambers were interconnected by a network of microfluidic channels. (b) The microfluidic analyzer (Dimension: 35 x 45 x 40 cm) comprises a fluidic actuation system, a thermal controlling system and an optical detection system.

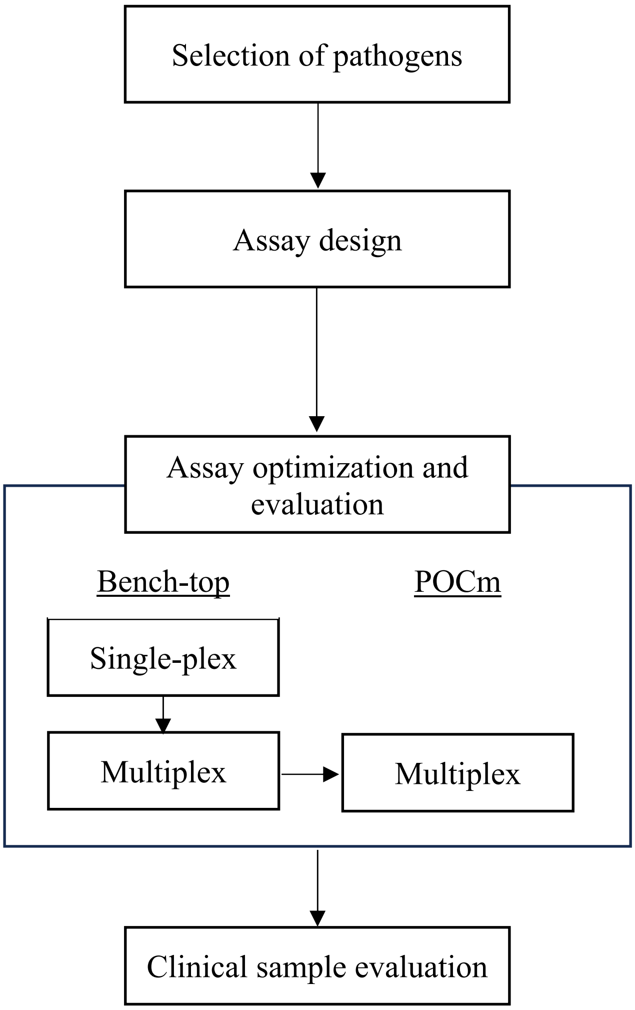

Selection of respiratory pathogens was advised by Department of Microbiology, The University of Hong Kong, based on clinical relevance and epidemiological significance in Hong Kong SAR and nearby regions.

The assay is based on nested real-time RT-PCR. It comprised a multiplexed one-step RT-PCR for 40 pathogen targets, sample quality controls (*GAPDH*) and processing control (*SUC1*). It was followed by an array of single-plex real-time PCRs for the pathogen targets, the two controls, and an additional real-time PCR control.

The assays were optimized by conventional manual methods using bench-top instruments individually, followed by multiplex reactions, which was then adopted to POCm. Evaluations on the analytical detectability of POCm included minimum detectable concentrations, cross-reactivity and linearity.

Clinical evaluation of POCm was carried out in the laboratory of Department of Microbiology, Queen Mary Hospital, Hong Kong, for a total of 283 archived samples. Sensitivity and specificity were calculated where appropriate.

Supplementary Figure S2. Schematic diagram of the current study.

Supplementary Table S1. Pathogen targets of the respiratory infectious disease assay panel

Pathogen	Abbreviation	Gene target(s)
Viruses:		
Adenovirus	AdV	*Hexon*
Bocavirus	BoV	*Non-structural protein-Viral capsid proteins (NP1-VP1)*
Coronavirus 229E	hCoV-229E	*Nucleocapsid (N) protein*
Coronavirus NL63	hCoV-NL63	*Nucleocapsid (N) protein*
Coronavirus HKU1	hCoV-HKU1	*Nucleocapsid (N) protein*
Coronavirus OC43	hCoV-OC43	*Nucleocapsid (N) protein*
Influenza A (Matrix)[a]	Flu A	*Matrix*
Influenza A (pdm09 H1)	Flu A/H1-2009	*Hemagglutinin*
Influenza A (H2)	Flu A/H2	*Hemagglutinin*
Influenza A (H3)	Flu A/H3	*Hemagglutinin*
Influenza A (H5)	Flu A/H5	*Hemagglutinin*
Influenza A (H6)	Flu A/H6	*Hemagglutinin*
Influenza A (H7)	Flu A/H7	*Hemagglutinin*
Influenza A (H9)	Flu A/H9	*Hemagglutinin*
Influenza A (H10)	Flu A/H10	*Hemagglutinin*
Influenza B	Flu B	*Matrix*
Influenza C	Flu C	*Matrix*
Middle East Respiratory Syndrome coronavirus	MERS-CoV	*Nucleocapsid (N) protein*
Metapneumovirus	hMPV	*Nucleoprotein (NP)*
Parainfluenza 1	hPIV 1	*Nucleoprotein (NP)*
Parainfluenza 2	hPIV 2	*Nucleoprotein (NP)*
Parainfluenza 3	hPIV 3	*Nucleoprotein (NP)*
Parainfluenza 4	hPIV 4	*Nucleoprotein (NP)*
Parechovirus	Parechovirus	*5'UTR*
Respiratory syncytial virus	RSV	*Nucleoprotein (NP)*
Severe acute respiratory syndrome coronavirus 2	SARS-CoV-2	*Envelope protein (E)*<br>*Nucleocapsid (N) protein*<br>(2 detection targets were used for SARS-CoV-2 virus)
Bacteria:		
Mycobacterium tuberculosis	*MTB*	*16S*
Mycoplasma pneumoniae	*MP*	*P1*
Legionella pneumophila	*LP*	*mip*
Bordetella pertussis	*B pertussis*	*Porinprotein*
Chlamydophila psittaci	*C psittaci*	*Major outermembrane protein*
Burkholderia pseudomallei	*BP-TAT*	*TAT*
Coxiella burnetii	*CB*	*IS1111*
Chlamydophila pneumoniae	*CP*	*16S*
Staphylococcus aureus	*SA-PVL*	*PVL*
Streptococcus pneumoniae	*SP*	*lytA*

Streptococcus pyogenes	*S pyogenes*	*spy*
Fungi:		
Cryptococcus neoformans/gattii	*CN*	*ITS*
Pneumocystis jiroveci	*PJ*	*SSU*

[a] Generic influenza A assay that detects all influenza A viruses regardless of strains.

Supplementary Table S2. Content of real-time PCR mini-chambers in POCm cartridge for respiratory disease pathogen detection.

Pathogen	No. of real-time PCR replicates (no. of mini-chambers[a])	Pathogen	No. of real-time PCR replicates (no. of mini-chambers[a])
AdV	3	hPIV 4	3
BoV	3	Parechovirus	3
hCoV-229E	3	RSV	3
hCoV-NL63	3	SARS-CoV-2/N	2
hCoV-HKU1	3	SARS-CoV-2/E	2
hCoV-OC43	3	*MTB*	2
Flu A	3	*MP*	3
Flu A/H1-2009	3	*LP*	3
Flu A/H2	3	*B pertussis*	2
Flu A/H3	3	*C psittaci*	3
Flu A/H5	3	*BP-TAT*	3
Flu A/H6	3	*CB*	3
Flu A/H7	3	*CP*	3
Flu A/H9	3	*SA-PVL*	3
Flu A/H10	3	*SP*	2
Flu B	3	*S pyogenes*	3
Flu C	3	*CN*	2
MERS-CoV	3	*PJ*	3
hMPV	3	*Quality controls:*	
hPIV 1	3	*GAPDH*	`2
hPIV 2	3	*SUC1*	2
hPIV 3	3	Real-time PCR control	2

[a] Total number of mini-chambers: 120

Supplementary Table S3. Construction of reference controls for evaluation tests.

Pathogen	Genetic material source	Reference control type
AdV	Plasmid (GenScript)	Plasmid
BoV	Plasmid (GenScript)	Plasmid
hCoV-229E	Genomic RNA (ATCC)	In-vitro transcribed RNA
hCoV-NL63	Plasmid (GenScript)	In-vitro transcribed RNA
hCoV-HKU1	Plasmid (GenScript)	In-vitro transcribed RNA
hCoV-OC43	Genomic RNA (ATCC)	In-vitro transcribed RNA
Flu A	Genomic RNA (ATCC)	In-vitro transcribed RNA
Flu A/H1-2009	Genomic RNA (ATCC)	In-vitro transcribed RNA
Flu A/H2	Plasmid (GenScript)	In-vitro transcribed RNA
Flu A/H3	Plasmid (GenScript)	In-vitro transcribed RNA
Flu A/H5	PCR control (Vircell)	In-vitro transcribed RNA
Flu A/H6	Plasmid (GenScript)	In-vitro transcribed RNA
Flu A/H7	Plasmid (GenScript)	In-vitro transcribed RNA
Flu A/H9	Plasmid (GenScript)	In-vitro transcribed RNA
Flu A/H10	Plasmid (GenScript)	In-vitro transcribed RNA
Flu B	Genomic RNA (ATCC)	In-vitro transcribed RNA
Flu C	Plasmid (GenScript)	In-vitro transcribed RNA
MERS-CoV	PCR control (Vircell)	In-vitro transcribed RNA
hMPV	Synthetic RNA (ATCC)	In-vitro transcribed RNA
hPIV 1	Genomic RNA (ATCC)	In-vitro transcribed RNA
hPIV 2	Genomic RNA (ATCC)	In-vitro transcribed RNA
hPIV 3	Genomic RNA (ATCC)	In-vitro transcribed RNA
hPIV 4	Genomic RNA (ATCC)	In-vitro transcribed RNA
Parechovirus	PCR control (Vircell)	In-vitro transcribed RNA
RSV	Genomic RNA (ATCC)	In-vitro transcribed RNA
SARS-CoV-2	Plasmid (in-house)	In-vitro transcribed RNA
MTB	PCR control (Vircell)	Plasmid
MP	Genomic DNA (ATCC)	Plasmid
LP	Genomic DNA (ATCC)	Plasmid
B pertussis	Genomic DNA (ATCC)	Plasmid
C psittaci	PCR control (Vircell)	Plasmid
BP-TAT	Genomic DNA (ATCC)	Plasmid
CB	PCR control (Vircell)	Plasmid
CP	Genomic DNA (ATCC)	Plasmid
SA-PVL	Plasmid (GenScript)	Plasmid
SP	Genomic DNA (ATCC)	Plasmid
S pyogenes	Genomic DNA (ATCC)	Plasmid
CN	Genomic DNA (ATCC)	Plasmid
PJ	Plasmid (GenScript)	Plasmid

Supplementary Table S4. Reference control mixtures constructed from reference controls of different pathogens.

Mix	No. of pathogen targets	Reference control materials included[a]
**1**	4	Flu A, Flu A/H1-2009, Flu A/H2, Flu A/H3
**2**	4	Flu A/H5, Flu A/H6, Flu A/H7, Flu A/H9
**3**	3	Flu A/H10, Flu B, Flu C
**4**	3	AdV, BoV, Parechovirus
**5**	4	hCoV-229E, hCoV-NL63, hCoV-OC43, hCoV-HKU1
**6**	3	MERS-CoV, SARS-CoV-2/N, SARS-CoV-2/E
**7**	4	hPIV 1, hPIV 2, hPIV 3, hPIV 4
**8**	4	RSV, hMPV, *MTB, B pertussis*
**9**	4	*C psittaci, BP-TAT, CB, CP*
**10**	4	*MP, LP, SP, S pyogenes*
**11**	3	*SA-PVL, PJ, CN,*
**12**	0	Negative control

[a] For all of the mixtures including negative control, total RNA from Hela cells was added to mimic background human RNA. The concentration of HeLa total RNA was equivalent to 50 cells per reaction, i.e., the amount that was usually observed in real human samples.

Supplementary Table S5. Information of pre-clinical study of POCm in Department of Microbiology, Queen Mary Hospital, Hong Kong

Supplementary Table S5a. Information of clinical specimens analysed by different versions of POCm assays

	**POCm version 1**	**POCm version 2**	**POCm version 3**
Real-time RT-PCR assay	11-plex (Supplementary Table S5b)	18-plex (Supplementary Table S5c)	40-plex (Supplementary Table S1)
Total no. of specimen	6	233	44
Nasopharyngeal aspirate	6	110	25
Nasopharyngeal swab	0	117	19
Combined nasopharyngeal and throat swab	0	1	0
Endotracheal aspirate	0	1	0
Deep throat saliva	0	4	0

Supplementary Table S5b. Assay content of 11-plex real-time RT-PCR in POCm version 1. Eleven pathogen strains were targeted by the assay.

**Pathogen**	**Gene target(s)**
Flu A	*Matrix*
Flu A/H1-2009	*Hemagglutinin*
Flu A/H2	*Hemagglutinin*
Flu A/H3	*Hemagglutinin*
Flu A/H5	*Hemagglutinin*
Flu A/H6	*Hemagglutinin*
Flu A/H7	*Hemagglutinin*
Flu A/H9	*Hemagglutinin*
Flu B	*Matrix*
SA-PVL	*PVL*
BP-TAT	*TAT*

Supplementary Table S5c. Assay content of 18-plex real-time RT-PCR in POCm version 2. Sixteen pathogen strains were targeted by the assay.

**Pathogen**	**Gene target(s)**
AdV	*Hexon*
Flu A	*Matrix*
Flu A/H1-2009	*Hemagglutinin*
Flu A/H3	*Hemagglutinin*
Flu B	*Matrix*
MERS-CoV	*Nucleoprotein (NP)*
hMPV	*Nucleoprotein (NP)*
hPIV 1	*Nucleoprotein (NP)*
hPIV 3	*Nucleoprotein (NP)*
RSV	*Nucleoprotein (NP)*

SARS-CoV-2	*Envelope protein (E)*<br>*Nucleocapsid phosphoprotein (N)*<br>*RNA dependent RNA polymerase (Rdrp)*
MP	*P1*
LP	*mip*
B pertussis	*Porinprotein*
CP	*16S*
S pyogenes	*spy*

Supplementary Table S6. Standard-of-care tests of the 39 selected pathogens in Department of Microbiology, Queen Mary Hospital, Hong Kong

Pathogen	Tests by Queen Mary Hospital[a]	Further tests by reference laboratory[b]
AdV	IFA / FilmArray	Respiratory multiplex PCR assay
BoV	In-house	-
hCoV-229E	In-house / FilmArray	-
hCoV-NL63	In-house / FilmArray	-
hCoV-HKU1	In-house / FilmArray	-
hCoV-OC43	In-house / FilmArray	-
Flu A	IFA / Xpert / FilmArray	Respiratory multiplex PCR assay
Flu A/H1-2009	In-house / FilmArray	Respiratory multiplex PCR assay
Flu A/H2	In-house	-
Flu A/H3	In-house / FilmArray	Respiratory multiplex PCR assay
Flu A/H5	In-house	-
Flu A/H6	In-house	-
Flu A/H7	In-house	-
Flu A/H9	In-house	-
Flu A/H10	In-house	-
Flu B	IFA / Xpert / FilmArray	Respiratory multiplex PCR assay
Flu C	In-house	Respiratory multiplex PCR assay
MERS-CoV	In-house	-
hMPV	IFA / FilmArray	Respiratory multiplex PCR assay
hPIV 1	IFA / FilmArray	Respiratory multiplex PCR assay
hPIV 2	IFA / FilmArray	Respiratory multiplex PCR assay
hPIV 3	IFA / FilmArray	Respiratory multiplex PCR assay
hPIV 4	In-house / FilmArray	Respiratory multiplex PCR assay
Parechovirus	In-house	-
RSV	IFA / Xpert / FilmArray	Respiratory multiplex PCR assay
SARS-CoV-2	Commercial kit	-
MTB	In-house	-
MP	In-house	-
LP	In-house / FilmArray	-
B pertussis	In-house	-
C psittaci	In-house / FilmArray	-
BP-TAT	In-house	-
CB	In-house	-
CP	In-house	-
SA-PVL	In-house / FilmArray	-
SP	In-house	-
S pyogenes	In-house	-
CN	In-house	-
PJ	In-house	-

[a] IFA: Immunofluorescent antibody assay (D3 Ultra 8 DFA Respiratory Virus Screening and ID Kit, QuidelOrtho)
FilmArray: FilmArray Respiratory Panel (bioMérieux)
Xpert: Xpert Xpress Flu/RSV (Cepheid)
In-house: In-house real-time RT-PCR for RNA virus; In-house real-time PCR for DNA virus, bacteria and fungi
Commercial kit for SARS-CoV-2: LightMix® Modular SARS and Wuhan CoV E-gene kit (TIB Molbiol, Berlin, Germany)

[b] For pathogens not covered by IFA and Xpert, further tests were conducted by reference laboratory (Public Health Laboratory Centre in Hong Kong). If the specimen was initially tested by FilmArray, no further tests are required

Supplementary Table S7. Clinical evaluation of the 39 pathogen targets separated by the three phases.

Target	Diagnosis	Version 1 (11-plex)			Version 2 (18-plex)			Version 3 (40-plex)			Combined version 1, 2 and 3		
		n	POCm result		n	POCm result		n	POCm result		n	POCm result	
			Positive (n)	Negative (n)		Positive (n)	Negative (n)		Positive (n)	Negative (n)		Positive (n)	Negative (n)
AdV	Positive	-	-	-	1	1	0	2	2	0	3	3	0
	Negative	-	-	-	233	0	233	41	0	41	274	0	274
BoV	Positive	-	-	-	-	-	-	0	0	0	0	0	0
	Negative	-	-	-	-	-	-	43	0	43	43	0	43
hCoV-229E	Positive	-	-	-	-	-	-	0	0	0	0	0	0
	Negative	-	-	-	-	-	-	43	0	43	43	0	43
hCoV-NL63	Positive	-	-	-	-	-	-	0	0	0	0	0	0
	Negative	-	-	-	-	-	-	43	0	43	43	0	43
hCoV-HKU1	Positive	-	-	-	-	-	-	0	0	0	0	0	0
	Negative	-	-	-	-	-	-	43	0	43	43	0	43
hCoV-OC43	Positive	-	-	-	-	-	-	0	0	0	0	0	0
	Negative	-	-	-	-	-	-	43	0	43	43	0	43
Flu A	Positive	6	6	0	34	29	5	9	9	0	49	44	5
	Negative	0	0	0	200	0	200	34	0	34	234	0	234
Flu A/H1-2009	Positive	6	6	0	28	26	2	8	8	0	42	40	2
	Negative	0	0	0	204	0	204	35	0	35	239	0	239
Flu A/H2	Positive	0	0	0	-	-	-	0	0	0	0	0	0
	Negative	6	0	6	-	-	-	43	0	43	49	0	49
Flu A/H3	Positive	0	0	0	6	6	0	1	1	0	7	7	0
	Negative	6	0	6	228	0	228	42	0	42	276	0	276
Flu A/H5	Positive	0	0	0	-	-	-	0	0	0	0	0	0
	Negative	6	0	6	-	-	-	43	0	43	49	0	49

Target	Diagnosis	Version 1 (11-plex)			Version 2 (18-plex)			Version 3 (40-plex)			Combined version 1, 2 and 3		
		n	POCm result		n	POCm result		n	POCm result		n	POCm result	
			Positive (n)	Negative (n)		Positive (n)	Negative (n)		Positive (n)	Negative (n)		Positive (n)	Negative (n)
Flu A/H6	Positive	0	0	0	-	-	-	0	0	0	0	0	0
	Negative	6	0	6	-	-	-	43	0	43	49	0	49
Flu A/H7	Positive	0	0	0	-	-	-	0	0	0	0	0	0
	Negative	6	0	6	-	-	-	43	0	43	49	0	49
Flu A/H9	Positive	0	0	0	-	-	-	0	0	0	0	0	0
	Negative	6	0	6	-	-	-	43	0	43	49	0	49
Flu A/H10	Positive	-	-	-	-	-	-	0	0	0	0	0	0
	Negative	-	-	-	-	-	-	43	0	43	43	0	43
Flu B	Positive	0	0	0	5	4	1	5	5	0	10	9	1
	Negative	6	0	6	229	0	229	38	0	38	273	0	273
Flu C	Positive	-	-	-	-	-	-	0	0	0	0	0	0
	Negative	-	-	-	-	-	-	43	0	43	43	0	43
MERS-CoV	Positive	-	-	-	0	0	0	0	0	0	0	0	0
	Negative	-	-	-	234	0	234	43	0	43	277	0	277
hMPV	Positive	-	-	-	1	1	0	4	4	0	5	5	0
	Negative	-	-	-	233	0	233	39	0	39	272	0	272
hPIV 1	Positive	-	-	-	3	3	0	5	5	0	8	8	0
	Negative	-	-	-	231	0	231	38	0	38	269	0	269
hPIV 2	Positive	-	-	-	-	-	-	0	0	0	0	0	0
	Negative	-	-	-	-	-	-	43	0	43	43	0	43
hPIV 3	Positive	-	-	-	7	7	0	4	4	0	11	11	0
	Negative	-	-	-	227	0	227	39	0	39	266	0	266
hPIV 4	Positive	-	-	-	-	-	-	2	2	0	2	2	0
	Negative	-	-	-	-	-	-	41	0	41	41	0	41

Target	Diagnosis	Version 1 (11-plex)			Version 2 (18-plex)			Version 3 (40-plex)			Combined version 1, 2 and 3		
		n	POCm result		n	POCm result		n	POCm result		n	POCm result	
			Positive (n)	Negative (n)		Positive (n)	Negative (n)		Positive (n)	Negative (n)		Positive (n)	Negative (n)
Parechovirus	Positive	-	-	-	-	-	-	0	0	0	0	0	0
	Negative	-	-	-	-	-	-	43	0	43	43	0	43
RSV	Positive	-	-	-	8	7	1	2	2	0	10	9	1
	Negative	-	-	-	226	0	226	41	0	41	267	0	267
SARS-CoV-2	Positive	-	-	-	19	17	2	5	5	0	24	22	2
	Negative	-	-	-	215	0	215	38	0	38	253	0	253
MTB	Positive	-	-	-	-	-	-	0	0	0	0	0	0
	Negative	-	-	-	-	-	-	43	0	43	43	0	43
MP	Positive	-	-	-	1	0	1	1	1	0	2	1	1
	Negative	-	-	-	233	0	233	42	0	42	275	0	275
LP	Positive	-	-	-	0	0	0	0	0	0	0	0	0
	Negative	-	-	-	234	0	234	43	0	43	277	0	277
B pertussis	Positive	-	-	-	0	0	0	0	0	0	0	0	0
	Negative	-	-	-	234	0	234	43	0	43	277	0	277
C psittaci	Positive	-	-	-	-	-	-	0	0	0	0	0	0
	Negative	-	-	-	-	-	-	43	0	43	43	0	43
BP-TAT	Positive	0	0	0	-	-	-	0	0	0	0	0	0
	Negative	6	0	6	-	-	-	43	0	43	49	0	49
CB	Positive	-	-	-	-	-	-	0	0	0	0	0	0
	Negative	-	-	-	-	-	-	43	0	43	43	0	43
CP	Positive	-	-	-	0	0	0	0	0	0	0	0	0
	Negative	-	-	-	234	0	234	43	0	43	277	0	277
SA-PVL	Positive	0	0	0	-	-	-	0	0	0	0	0	0
	Negative	6	0	6	-	-	-	43	0	43	49	0	49

Target	Diagnosis	Version 1 (11-plex)			Version 2 (18-plex)			Version 3 (40-plex)			Combined version 1, 2 and 3		
		n	POCm result		n	POCm result		n	POCm result		n	POCm result	
			Positive (n)	Negative (n)		Positive (n)	Negative (n)		Positive (n)	Negative (n)		Positive (n)	Negative (n)
SP	Positive	-	-	-	-	-	-	2	2	0	2	2	0
	Negative	-	-	-	-	-	-	41	0	41	41	0	41
S pyogenes	Positive	-	-	-	0	0	0	0	0	0	0	0	0
	Negative	-	-	-	234	0	234	43	0	43	277	0	277
CN	Positive	-	-	-	-	-	-	0	0	0	0	0	0
	Negative	-	-	-	-	-	-	43	0	43	43	0	43
PJ	Positive	-	-	-	-	-	-	0	0	0	0	0	0
	Negative	-	-	-	-	-	-	43	0	43	43	0	43
